# Metal Nanoparticles Formation from Nickel Hydroxide

**DOI:** 10.3390/ma13204689

**Published:** 2020-10-21

**Authors:** Elena N. Sidorova, Ella L. Dzidziguri, Yulia P. Vinichenko, Dmitriy Yu. Ozherelkov, Alexander S. Shinkaryov, Alexander A. Gromov, Anton Yu. Nalivaiko

**Affiliations:** 1 Department of Functional Nanosystems and High–Temperature Materials, National University of Science and Technology MISIS, 119991 Moscow, Russia; sidelen@mail.ru (E.N.S.); avrore@gmail.com (E.L.D.); julinadinno@gmail.com (Y.P.V.); 2 Department of Metallurgical Science & the Physics of Strength, National University of Science and Technology MISIS, 119991 Moscow, Russia; d.ozherelkov@gmail.com; 3 MISIS Catalysis Lab, National University of Science and Technology MISIS, 119991 Moscow, Russia; shinkaryov@gmail.com (A.S.S.); a.gromov@misis.ru (A.A.G.)

**Keywords:** nanoparticles, nanotechnology, characterization, reduction, hydroxide, nickel, XRD, Raman spectroscopy

## Abstract

In this study, the mechanism of nickel nanoparticle formation from its hydroxide was analyzed. Metallic nickel nanoparticles were obtained through the hydroxide’s reduction under hydrogen. Nickel hydroxides were produced from nickel (II) nitrate hexahydrate and NaOH by deposition under various initial conditions. The influence of washing treatment on the dispersion of obtained nickel powders was studied. The washing procedure of precipitates was carried out by centrifugation, ultrasonic treatment, and decantation. X-ray diffractometry, transmission electron microscopy, low-temperature nitrogen adsorption, infrared spectroscopy, Raman spectroscopy, and X-ray photoelectron spectroscopy methods were used for nanoparticle characterization. Based on the resulting data, a model of the Ni(OH)_2_ aggregate structure after deposition was proposed. The number of nickel hydroxide particles required to form one nickel nanoparticle was estimated, and a model of its formation was proposed.

## 1. Introduction

In the last decade, the preparation and use of nanomaterials have been two of the most promising areas of scientific research due to the unique properties of nano-sized structural components [1,2,3]. Nanomaterials with different morphologies (particles, films, and tubes) are of great research interest for various applications in areas such as microelectronics, medicine, catalysis, energy, etc. [4,5,6].

Nickel-based nanoscale powders have attracted the interest of researchers due to their good catalytic, magnetic, and tribological properties [7,8,9,10]. They are used as additives in motor oils, in storage systems, in magnetic cooling systems, in catalysis [11,12,13], and as permanent magnets [14,15]. Several methods have been developed to produce nickel nanoparticles, among which are chemical control reduction, the ethanol-water system, the polyol method, hydrazine reduction, solution reduction, laser ablation synthesis in solution (LASIS), and ultrasound reduction. These techniques are low cost and require simple equipment. The ethanol-water system method can be conducted at room temperature with very cheap reagents. The ultrasound reduction method can accelerate and improve the reaction efficiency by ultrasonic cavitation energy, while in the polyol process, the polyol itself can act as a protective agent to avoid particle agglomeration and growth. Hydrazine reduction is another synthetic route without any other protective agent and inert atmosphere protection performed at room temperature. The hydrothermal reduction method allows the straightforward preparation of pure nickel nanoparticles when pH ≥ 10.0 and T ≥ 85 °C. The advantages of nanoparticles obtained by non-chemical methods (for example, the LASIS technique) are the higher purity and stability of produced nanoparticles [16,17]. Among all the magnetic metallic nanomaterials, nickel nano-structure materials are difficult to prepare because they are easily oxidizable. Therefore, the majority of these methods have shown several disadvantages, including harsh reaction conditions, easy agglomeration, and the oxidation of specimens. Although the methods mentioned above have their benefits and drawbacks, the chemical methods are still largely used and have been extended as they offer good control of the particle size and morphology. Additionally, chemical methods are suitable for possible bulk factory production in further attempts.

Currently, the lack of understanding of a new phase formation mechanism is one of the major problems in the control of nanomaterial dispersion. The reduction process is used for obtaining metallic nanoparticles, with the “shrinking core” being the most common model used to describe the formation mechanism of the metal phase during the reduction process [18,19,20]. According to the “shrinking core” model, the chemical reaction zone moves frontally deep, into the initial phase [21,22,23,24]. The reduction begins on the surface of the particle, and until the outer layer is completely transformed into the corresponding reaction products, the inner parts of the material do not react. Gradually, the chemical reaction zone moves deeper into the particle, leaving the solid reaction products behind. In particular, during the reduction process, these products can be lower oxides or the metal itself. Therefore, at some arbitrary reduction moment, the particle is the inner core of the initial substance, surrounded by one or several shells of the reaction products.

The current research investigates an application of the “shrinking core” mechanism to nanomaterial formation. Metallic nanoparticles have been objects of research for many years, but the processes that occur during their formation are still understudied. Though there is a multitude of chemical methods available for nanoparticle production, the issue relating to dispersion and particle shape control is still unsettled. Hence, the purpose of this research is to determine the mechanism of nickel nanoparticle formation and its influence on their dispersion. The synthesized nickel nanoparticles are considered an addition to metal powders for additive manufacturing. Three-dimensional printing, as a frontier manufacturing technique, enables the rapid production of complex geometry components with unique and highly tailorable microstructures [25,26]. However, one of the key problems in additive manufacturing is the limited amount of materials suitable for 3D printing. Metal matrix composites with secondary metal particles as additions represent one of the solutions for eliminating this issue and allow the fabrication of materials with a reduced grain size and improved mechanical properties [27].

## 2. Materials and Methods 

The original reactants used for nickel hydroxide production were nickel (II) nitrate hexahydrate (analytical grade) and NaOH alkali (analytical grade). The nickel hydroxide powder was obtained by a chemical deposition method using the NANOHIM hardware with a software complex. The setting of the experiment parameters, the control of all the devices, and the recording of the results were automated.

During the chemical process and the nickel hydroxide sample preparation [28,29], the temperature was varied. Thermostatic control using a Lauda E 300 (Lauda Dr. R. Wobser GmbH & Co. KG, Lauda-Königshofen, Germany) thermostat was carried out at temperatures of 15, 20, 30, and 45 °C, with silicone oil as a working fluid. The pH values varied between 8 and 10 and were measured with a Mettler Toledo MP 230 pH meter. (Mettler-Toledo (HK) MTCN Limtied, Hong Kong). 

The deposition was carried out in the reactor by the dosed supply of salt solutions and a precipitator with continuous stirring. The deposition temperature was maintained by the thermostat and the acidity was controlled with a pH meter. The obtained deposits were washed with distilled water and dried. Washing of the deposits was carried out by two methods: Centrifugation using a high-speed Hettich Rotanta 460 (Andreas Hettich GmbH & Co. KG, Tuttlingen, Germany) centrifuge (centrifugation was followed by dispersion in distilled water using a high-intensity ultrasonic treatment and subsequent centrifugation) and decantation. Additionally, ultrasonic treatment was carried out on one of the samples using a Hielscher UIP 1000hd (Hielscher USA, Inc., Wanaque, NJ, USA) ultrasonic homogenizer to divide the aggregates during the deposition process. A Snol 58/350 (AB “UMEGA GROUP”, Utena, Lithuania) drying oven was employed to dry the obtained precipitate of the nickel hydroxide. 

**Figure 1:**
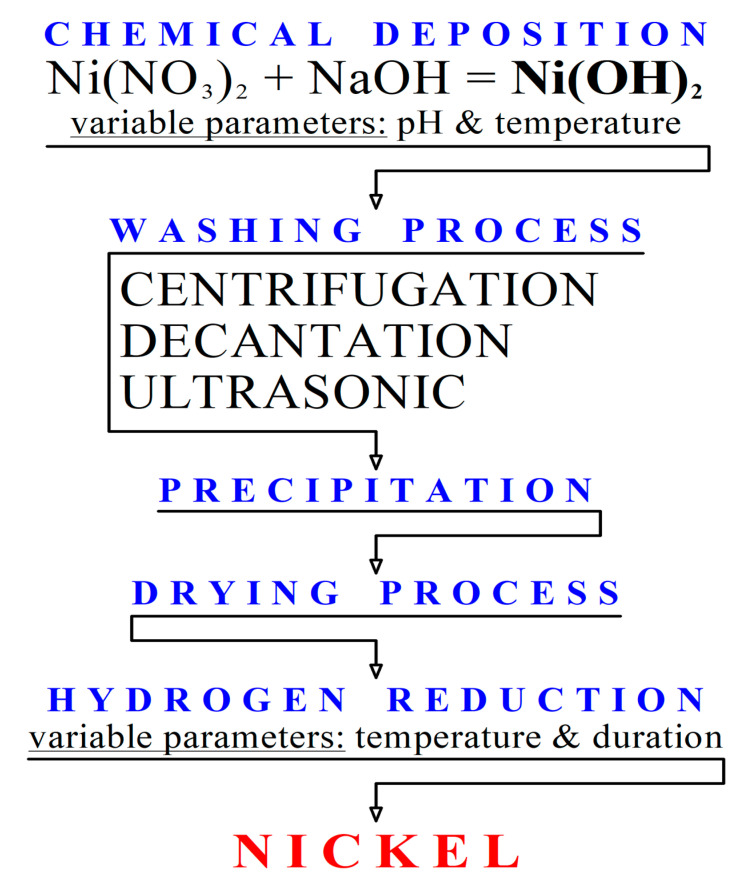
Scheme for the production of the nickel nanoparticles.

Solutions were pre-prepared with a given concentration, based on the required volume of the nano-sized product. The metallic nickel samples were obtained from the nickel hydroxides at various temperatures (200, 220, and 280 °C) and reduction times (15, 30, 45, 60, 90, and 120 min). A technological scheme of the nickel nanoparticles obtained is shown in Figure 1. The synthesis conditions of the samples and their designations are presented in Table 1.

The phase composition of powders before and after the reduction was studied by a Difrey-401 X-ray(Scientific Instruments, JSC, Saint Petersburg, Russia) diffractometer using Cr kα radiation. The sizes of the coherent scattering regions were calculated for the (100) and (101) planes of the nickel hydroxide using the Scherrer equation. The microstructure and morphology of the obtained samples were analyzed using a TEM LEO 912 AB (Zeiss) (Carl Zeiss, Jena, Germany) and a Tescan Vega 3 SEM. (Tescan Analytics, Fuveau, France).

Specific surface area values for all the nickel hydroxide samples were obtained using the NOVA 1200e (Quantachrome, Boynton Beach, FL, USA) analyzer by a low-temperature nitrogen adsorption method. The size of the aggregates from the experimental data under the assumption of their sphericity was calculated by Equation (1):

(1) $$D\;=\;\frac{6}{{\rho \;\times \;S}_{\mathrm{sp}}}$$

where D is the diameter of the aggregate, m; ρ is the density of the nickel hydroxide, kg/m^3^; and S_sp_ is the specific area, m^2^/kg.

Verification of the specific surface area and dispersion characteristics obtained by electron microscopy was based on the cylindrical shape of the particles and was calculated by the following ratio (2):

(2) $$S_{\mathrm{sp}}\;=\frac{2d\;+\;4h}{d\;\times \;h\;\times \;\rho }$$

where S_sp_ is the specific area, m^2^/kg; d is the cylinder base diameter, m; h is the cylinder height, m; and ρ is the density, kg/m^3^.

The number of nickel hydroxide plates (N) was estimated by Equation (3):

(3) N = Vaggregate Vplate = π × D36π × d2 × h4 = 2D33d2 × h

where D is the diameter of the aggregate, m, and d is the diameter of the plate, m.

The IR spectra were obtained by an Attenuated Total Reflectance (ATR) method using a Thermo Nicolet 380 (Thermo Fisher Scientific, Waltham, MA, USA) spectrometer with a Smart iTR attachment. Each spectrum was obtained by the averaging of 32 mirror passes. The comparison spectrum was initially collected for each sample.

The chemical and electronic states of atoms in the samples were determined using a PHI5500 VersaProbe II X-ray (Physical Electronics, Inc., Chanhassen, MN, USA) photoelectron spectrometer. Monochromatized Al Kα radiation (hν = 1486.6 eV) at a 50 W power was used as the excitation source. The binding energy of the O1s photoelectron line was determined from the high-resolution spectra taken at an analyzer transmittance of 23.5 eV and a data collection density of 0.2 eV/step. The spectra were approximated by a nonlinear least-squares method using the Gauss‐Lorentz function. The binding energy scale was calibrated by the C1s spectrum of adsorbed hydrocarbons at 285.0 eV. The binding energies determination error was ±0.3 eV.

## 3. Results and Discussion

**Figure 2:**
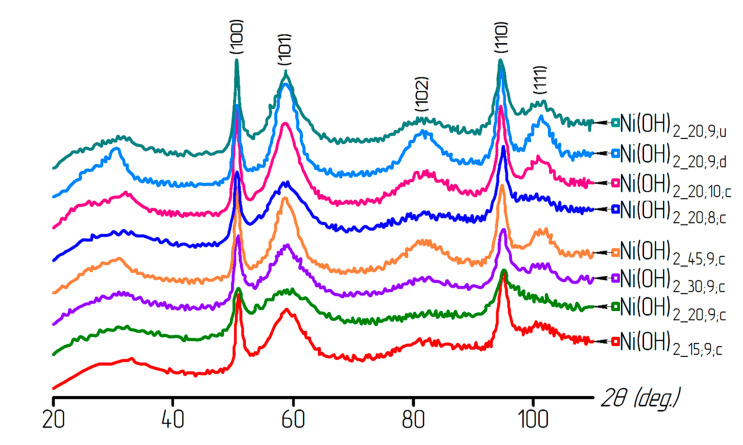
X-ray patterns of the nickel hydroxide samples obtained under various deposition conditions.

Based on X-ray analysis, all nickel hydroxide samples were shown to consist of the Ni(OH)_2_ phase (α-modification of the nickel hydroxide (α-Ni(OH)_2_·xH_2_O) with a hexagonal lattice. The X-ray diffraction patterns of the samples are presented in Figure 2.

The calculation results of the nickel hydroxide lattice periods are presented in Table 2. From the data obtained, it can be seen that the values of a and c parameters differ significantly from the reference values. Such differences may occur due to the Ni(OH)_2_ lattice structure being deformed by the presented molecules of interlayer water.

**Figure 3:**
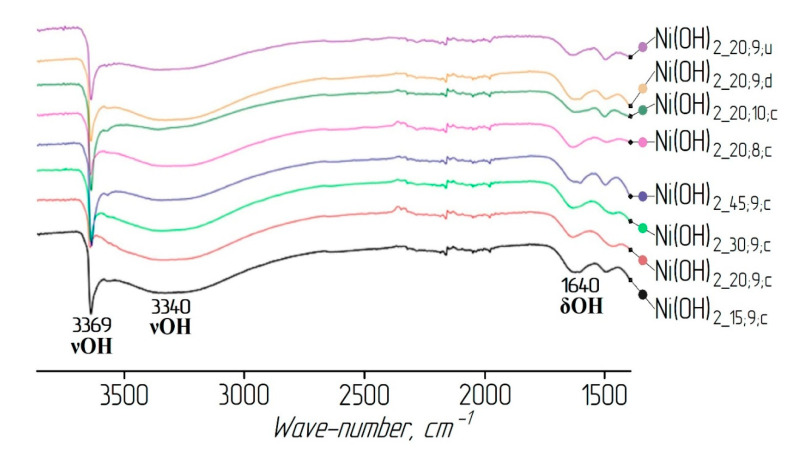
IR spectra of the nickel hydroxide samples.

**Figure 4:**
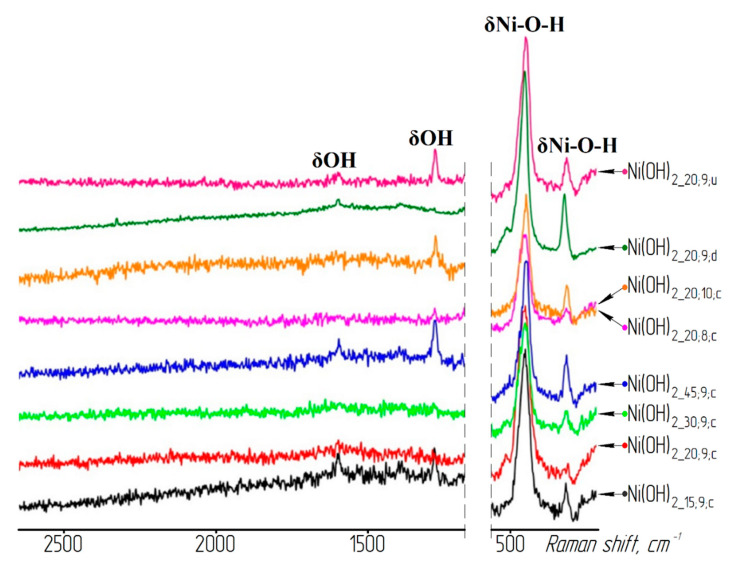
Fragments of the nickel hydroxide samples’ Raman spectra.

The presence of water molecules in the nickel hydroxide samples was confirmed by the results of IR and Raman spectroscopy (see Figure 3 and Figure 4). A wide absorption band in the region of wave numbers between 3600 and 3200 cm^−1^ intrinsic to OH group stretching vibrations in the interlayer water molecules [30] and the bands in the region between 1600 and 1650 cm^−1^ correspond to the deformation vibrations of the water molecule hydroxyl groups.

**Figure 5:**
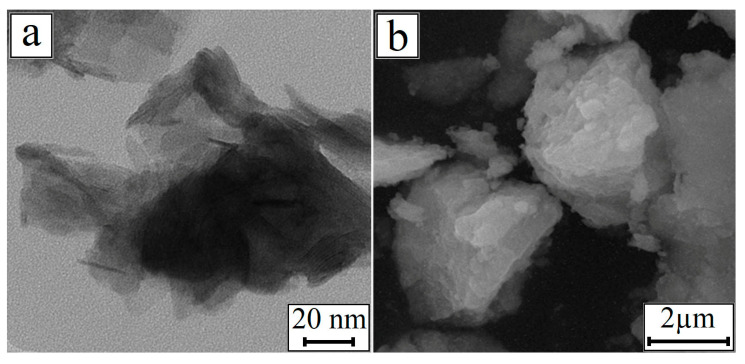
Nickel hydroxide microstructure: (**a**) TEM of Ni(OH)_2__20;10;c and (**b**) SEM of Ni(OH)_2__20;9;u.

The microstructure of the nickel hydroxide is shown in Figure 5. TEM and SEM images showed that the nickel hydroxide particles in all of the samples were collected in aggregates, regardless of the preparation conditions. The particles represented thin cylindrical plates with a height of several nanometers. Therefore, their diameter was specified as the particle size.

Knowing the diameter of the aggregate, one can calculate its volume and number of nickel hydroxide plates (N) using Equation (3). The average particle diameters and the specific surface area for all of the nickel hydroxide samples were determined and are presented in Table 3.

The calculated data presented in Table 3 do not fit the results obtained by electron microscopy. If the values of the diameter and the thickness of the film are used in Equation (1), the value of the specific surface area should be two orders of magnitude greater. This difference occurs due to the fact that hydroxide particles are highly aggregated.

**Figure 6:**
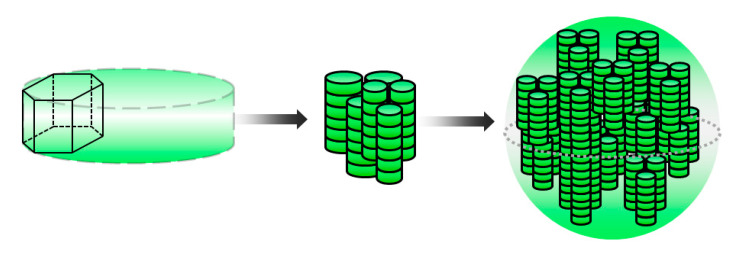
Nickel hydroxide particle aggregation scheme.

Based on the obtained values of the dimensional characteristics, it was possible to estimate how many particles were in an average aggregate in each sample of the nickel hydroxide. For simplicity, we assumed that the units were cylindrical (Figure 6). In this case, the size of the unit was calculated by Equation (2). As can be seen from the obtained results, the samples of the nickel hydroxide form aggregates from a completely different number of particles. This may be due to the various synthesis conditions employed. For example, the use of decantation allows the development of small aggregates. Centrifugation and even pre-treatment with ultrasound during the deposition process do not allow the dispersion of the material to be preserved.

**Figure 7:**
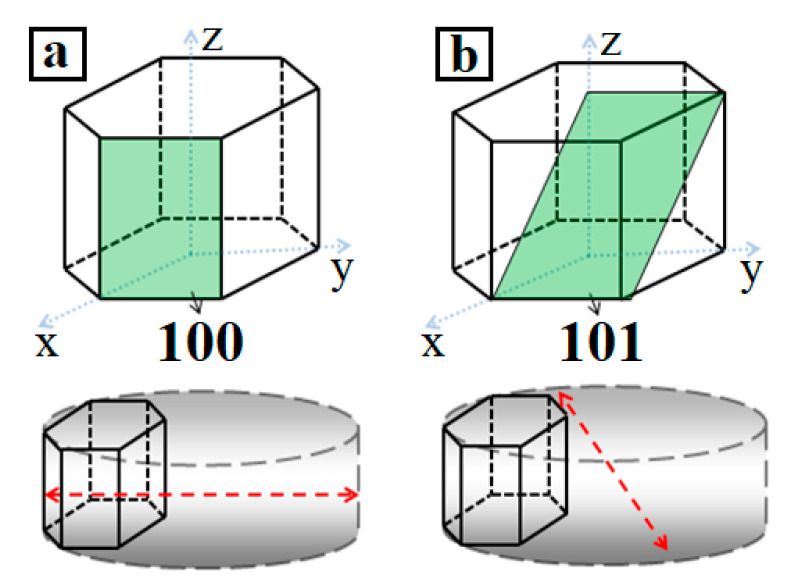
Coherent scattering region (CSR) sizes for the (100) (**a**) and (101) (**b**) planes.

The coherent scattering region (CSR) was determined in the direction perpendicular to the reflection planes. If the crystal is oriented as shown in Figure 7a, then the plate diameter can be determined by the broadening of the diffraction maximum from the (100) plane (red line in Figure 7a). Therefore, it is possible to estimate the thickness of the cylinder (Figure 7b) by using the size of the CSR calculated along the (101) plane.

The CSR sizes for all of the nickel hydroxide samples are presented in Table 4. For comparison, the average particle sizes, calculated from the microphotographs, are also given. As can be seen from the obtained data, the CSR sizes from the (100) plane are comparable to the particle diameters and the CSR sizes from the (101) plane are comparable to their thickness. These results suggest that, during deposition, the hydroxide particles form a crystal with a base along the (001) plane and a height along the *Z*-axis of the hexagonal lattice.

Table 4 shows that the CSR sizes for some samples along the (100) plane were larger than the average sizes calculated from electron microscopy. This was caused by the polydispersity of nickel hydroxide powders.

**Figure 8:**
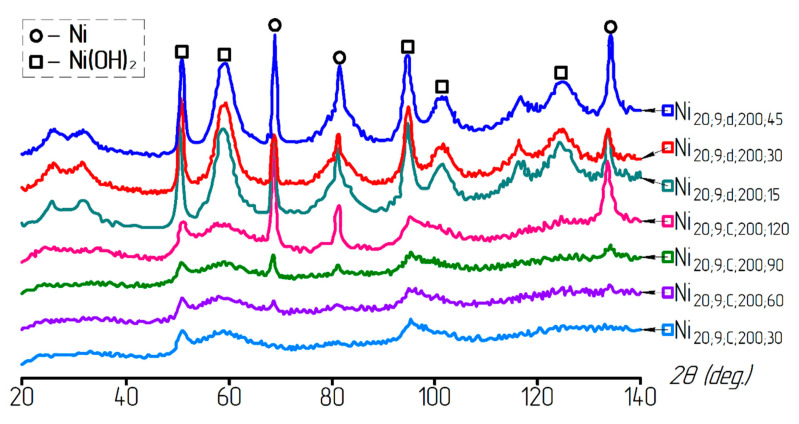
XRD patterns of the samples after reduction at a temperature of 200 °C.

Figure 8 presents the X-ray diffraction patterns of the samples reduced under various conditions. A metallic nickel powder was obtained from a hydroxide at a temperature of 200 °C. The analysis of the X-ray diffraction patterns showed that, for the samples washed in a centrifuge, the nickel phase appeared as a result of 60 min reduction at a temperature of 200 °C, whereas, after decantation, sufficiently large peaks of the nickel phase were detected after 15 min of reduction at the same temperature. It should also be noted that the X-ray analysis did not reveal a nickel oxide phase. This could be due to either a small amount of oxide (<5%), as the equipment sensitivity did not allow this phase to be fixed, or the formation of nickel particles directly from the hydroxide phase.

**Figure 9:**
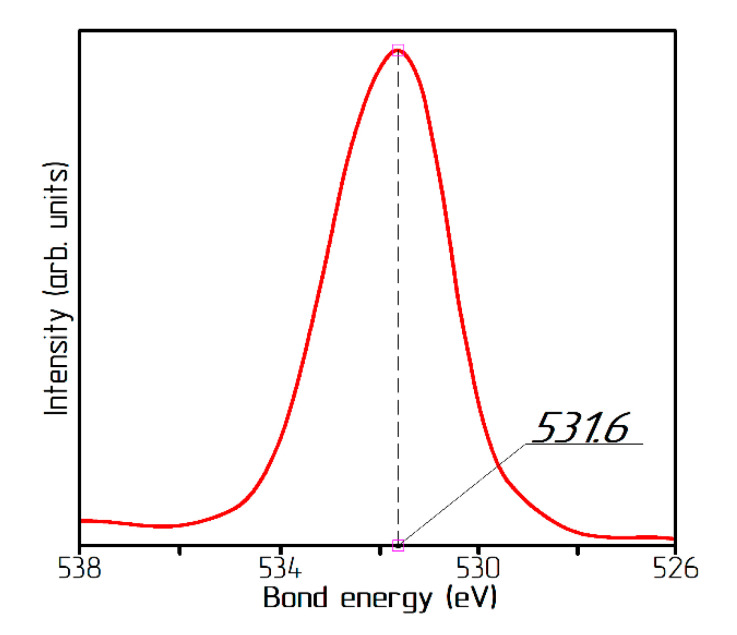
O1s spectrum.

The presence of the NiO oxide in the samples could be detected by X-ray photoelectron spectroscopy by the peak in the O1s spectrum in the region of 532.6 eV [31]. In the studied samples, the O1s spectrum had a maximum at a bond energy E = 531.6 eV (Figure 9), which corresponds to the oxygen in the Ni(OH)_2_ hydroxide. In the form of the spectrum, there were no obvious twists or influxes for objective approximation, which indicated the absence of oxygen in the energy state corresponding to NiO molecules. Based on the fact that even individual molecules of nickel oxide were not detected, the process of hydrogen reduction of the α-modification of the nickel hydroxide proceeded without the formation of a nickel oxide phase.

**Figure 10:**
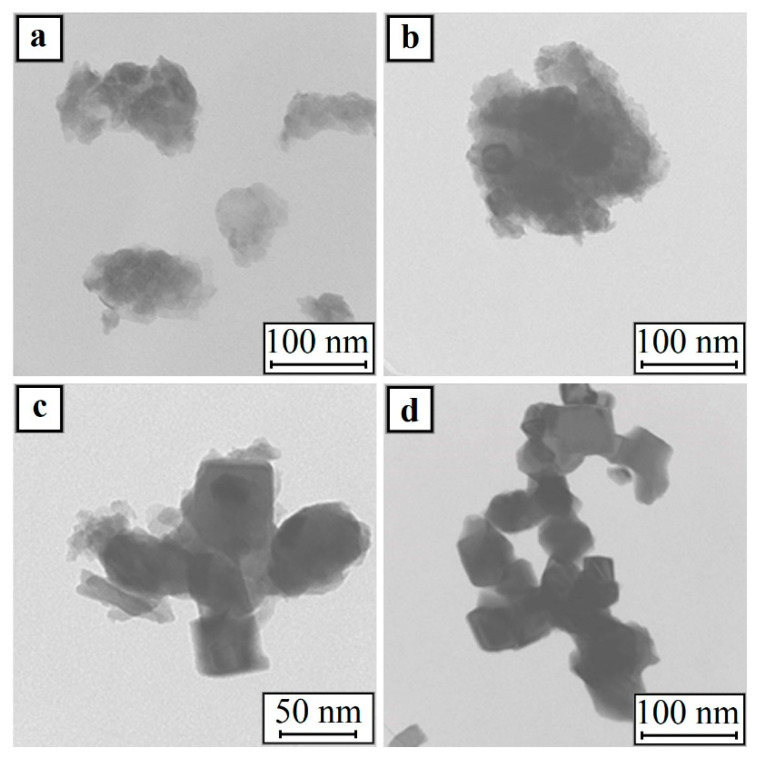
TEM images of the samples: (**a**) Ni_20;9;d;200;45_; (**b**) Ni_20;9;c;200;90_; (**c**) Ni_20;9;d;220;30_; and (**d**) Ni_20;9;d;280;15._

**Figure 11:**
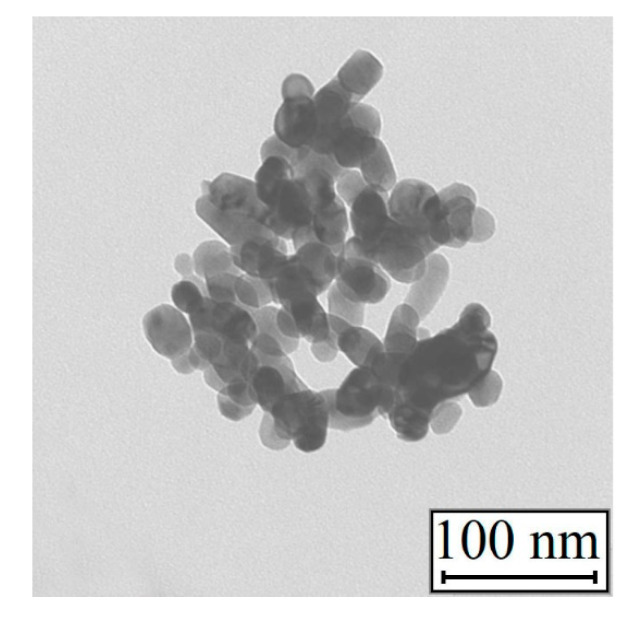
TEM images of the nickel oxide.

The TEM images of the samples after partial reduction (Figure 10a,b) showed that, in addition to the films of the initial nickel hydroxide, the material contains cubic particles of metallic nickel. The presence of a metal phase in the samples was determined by the X-ray diffraction analysis. The nickel oxide phases, as shown above, were not detected in the nanoparticles. In addition, after dehydration, NiO had a spherical shape (Figure 11).

The nickel hydroxide fully reduced within 15 min at 280 °C (sample: Ni_20;9;d;280;15_). At the same time, the nickel nanoparticles retained the shape of a cube (see Figure 10d). These results suggest that the metal nickel nanoparticles formed have a cubic shape.

The calculation of the average size of partially reduced Ni(OH)_2___20;9;c;280;15_ powder showed that the size of the cube edge was 17 nm. The average cube edge length of the nickel particles in the all-metal sample was about 28 nm.

After measurement of the Ni(OH)_2_ and Ni average sizes, an estimation of the number of nickel hydroxide plates in one nickel particle was made. The calculation results are presented in Table 5.

**Figure 12:**
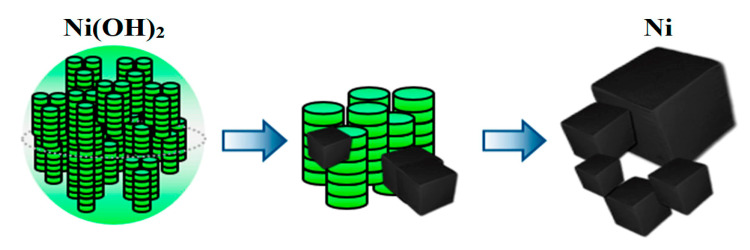
Scheme of metallic nickel formation from its hydroxide.

Therefore, one nickel particle with a 28 nm cube edge is formed from about 150 nickel hydroxide plates. According to the obtained results, the pattern of nickel nanopowder formation is shown in Figure 12.

The growth of the nickel phase occurs outside, rather than inside, of the nickel hydroxide aggregate. The morphologies of the source material and the reduced product are completely different. The obtained results allow us to conclude that the formation of nickel nanoparticles from their hydroxide does not follow the “shrinking core” mechanism.

## 4. Conclusions

Regardless of the deposition and washing conditions, alpha modification of the nickel hydroxide with a hexagonal lattice was observed. The obtained hydroxide was an aggregate consisting of thin plates successively stacked on top of each other. The aggregate had a cylindrical shape of about a 1 nm height, with a base along the (001) plane and a height along the *Z*-axis. Based on the obtained data, an estimated calculation of the number of plates forming individual aggregates was carried out. The calculation showed that their content varied from 28 to 3 × 10^5^ plates in one aggregate.

Various washing conditions lead to the formation of hydroxide with various dispersions. Decantation reduced the size of the aggregates and centrifugation reduced dispersion, evening out the effect of grinding by an ultrasonic treatment during the deposition process.

Low-temperature reduction allowed us to obtain unreduced samples containing phases of both metallic nickel and nickel hydroxide. At the same time, the nickel oxide phase was not detected by X-ray analysis. The absence of the NiO phase was confirmed by photoelectron spectroscopy.

The TEM studies of the two-phase samples, the metallic nickel nanopowder, and the dehydrated hydroxide (NiO), along with theoretical calculations, allowed us to make assumptions on the formation pattern of metallic nickel from its hydroxide during hydrogen reduction.

## Figures and Tables

**Table 1:** Synthesis conditions of the nickel hydroxide and nickel samples.

Sample	T, °C	pH	Washing	Reduced Sample	T, °C	Time, min
Ni(OH)_2__15;9;c	15	9	centrifuge	-	-	-
Ni(OH)_2__20;9;c	20	9	centrifuge	Ni_20;9;c;200;30_	200	30
				Ni_20;9;c;200;60_	200	60
				Ni_20;9;c;200;90_	200	90
				Ni_20;9;c;200;120_	200	120
Ni(OH)_2__30;9;c	30	9	centrifuge	-	-	-
Ni(OH)_2__45;9;c	45	9	centrifuge	-	-	-
Ni(OH)_2__20;8;c	20	8	centrifuge	-	-	-
Ni(OH)_2__20;10;c	20	10	centrifuge	-	-	-
Ni(OH)_2__20;9;d	20	9	decantation + centrifuge	Ni_20;9;d;200;15_	200	15
				Ni_20;9;d;200;30_	200	30
				Ni_20;9;d;200;45_	200	45
				Ni_20;9;d;220;30_	220	30
Ni(OH)_2__20;9;u	20	9	ultrasound + centrifuge	-	-	-

**Table 2:** Lattice periods of the nickel hydroxide samples.

Sample	a, nm	c, nm
Reference values	0.3117	0.4595
Ni(OH)_2__15;9;c	0.3071	0.4790
Ni(OH)_2__20;9;c	0.3082	0.4762
Ni(OH)_2__30;9;c	0.3077	0.4731
Ni(OH)_2__45;9;c	0.3091	0.4816
Ni(OH)_2__20;8;c	0.3088	0.4737
Ni(OH)_2__20;10;c	0.3094	0.4786
Ni(OH)_2__20;9;d	0.3089	0.4762
Ni(OH)_2__20;9;u	0.3095	0.4796

**Table 3:** Calculated parameters of the nickel hydroxide aggregate.

Sample	S_sp_, m^2^/g	Average Aggregate Diameter, nm	Plate Diameter, nm	Number of Plates in One Aggregate, N
Ni(OH)_2__15;9;c	8	183	25	5230
Ni(OH)_2__20;9;c	3	472	17	312,073
Ni(OH)_2__30;9;c	13	109	17	2985
Ni(OH)_2__45;9;c	52	27	24	28
Ni(OH)_2__20;8;c	5	264	15	37,697
Ni(OH)_2__20;10;c	35	41	18	164
Ni(OH)_2__20;9;d	39	36	25	61
Ni(OH)_2__20;9;u	4	400	21	80,481

**Table 4:** CSR sizes and particle sizes calculated from the electron microscopy results.

Sample	CSR Sizes, nm		Base Diameter TEM, nm
	(100)	(101)	
Ni(OH)_2__15;9;c	39	4	28
Ni(OH)_2__20;9;c	15	5	15
Ni(OH)_2__30;9;c	34	8	17
Ni(OH)_2__45;9;c	30	6	22
Ni(OH)_2__20;8;c	25	5	18
Ni(OH)_2__20;10;c	29	5	17
Ni(OH)_2__20;9;d	36	5	23
Ni(OH)_2__20;9;u	19	4	23

**Table 5:** Estimation of the number of nickel atoms in the samples.

Sample	Ni(OH)_2_	Ni
Average size, nm	base diameter 38.5	cube edge 28
Volume, nm^3^	29,880	22,222
Nickel atoms number	12,944	2,020,224
